# Selective publication of antidepressant trials and its influence on apparent efficacy: Updated comparisons and meta-analyses of newer versus older trials

**DOI:** 10.1371/journal.pmed.1003886

**Published:** 2022-01-19

**Authors:** Erick H. Turner, Andrea Cipriani, Toshi A. Furukawa, Georgia Salanti, Ymkje Anna de Vries

**Affiliations:** 1 Behavioral Health and Neurosciences Division, Veterans Affairs Portland Health Care System, Portland, Oregon, United States of America; 2 Department of Psychiatry, Oregon Health & Science University, Portland, Oregon, United States of America; 3 Department of Psychiatry, University of Oxford, Oxford, United Kingdom; 4 Oxford Health NHS Foundation Trust, Warneford Hospital, Oxford, United Kingdom; 5 Oxford Precision Psychiatry Lab, NIHR Oxford Health Biomedical Research Centre, Oxford, United Kingdom; 6 Department of Health Promotion and Human Behavior, Kyoto University Graduate School of Medicine/School of Public Health, Kyoto, Japan; 7 Institute of Social and Preventive Medicine, University of Bern, Bern, Switzerland; 8 Department of Psychiatry, Interdisciplinary Center Psychopathology and Emotion Regulation, University Medical Center Groningen, University of Groningen, Groningen, the Netherlands; 9 Developmental Psychology, Department of Psychology, University of Groningen, Groningen, the Netherlands; Harvard University, Brigham and Women’s Hospital, UNITED STATES

## Abstract

**Background:**

Valid assessment of drug efficacy and safety requires an evidence base free of reporting bias. Using trial reports in Food and Drug Administration (FDA) drug approval packages as a gold standard, we previously found that the published literature inflated the apparent efficacy of antidepressant drugs. The objective of the current study was to determine whether this has improved with recently approved drugs.

**Methods and findings:**

Using medical and statistical reviews in FDA drug approval packages, we identified 30 Phase II/III double-blind placebo-controlled acute monotherapy trials, involving 13,747 patients, of desvenlafaxine, vilazodone, levomilnacipran, and vortioxetine; we then identified corresponding published reports. We compared the data from this newer cohort of antidepressants (approved February 2008 to September 2013) with the previously published dataset on 74 trials of 12 older antidepressants (approved December 1987 to August 2002).

Using logistic regression, we examined the effects of trial outcome and trial cohort (newer versus older) on transparent reporting (whether published and FDA conclusions agreed). Among newer antidepressants, transparent publication occurred more with positive (15/15 = 100%) than negative (7/15 = 47%) trials (OR 35.1, CI_95%_ 1.8 to 693). Controlling for trial outcome, transparent publication occurred more with newer than older trials (OR 6.6, CI_95%_ 1.6 to 26.4). Within negative trials, transparent reporting increased from 11% to 47%.

We also conducted and contrasted FDA- and journal-based meta-analyses. For newer antidepressants, FDA-based effect size (ES_FDA_) was 0.24 (CI_95%_ 0.18 to 0.30), while journal-based effect size (ES_Journals_) was 0.29 (CI_95%_ 0.23 to 0.36). Thus, effect size inflation, presumably due to reporting bias, was 0.05, less than for older antidepressants (0.10).

Limitations of this study include a small number of trials and drugs—belonging to a single class—and a focus on efficacy (versus safety).

**Conclusions:**

Reporting bias persists but appears to have diminished for newer, compared to older, antidepressants. Continued efforts are needed to further improve transparency in the scientific literature.

## Author summary

**Why was this study done?:**

➢ Clinicians and researchers depend on the peer-reviewed literature for accurate assessments of drug efficacy and safety, but this depends on whether the outcomes of all trials—negative, as well as positive—are reported transparently.
➢ In an earlier study, using Food and Drug Administration (FDA) review documents as a gold standard, we found that many negative trials had been misreported in the published literature as having positive outcomes or had simply not been published.
➢ Since then, reporting bias has been the subject of additional studies and policy changes, raising the question, Is the antidepressant literature now being reported more transparently?

**What did the researchers do and find?:**

➢ Using FDA reviews on 4 newer antidepressants, we identified 30 trials, half with positive, and half with negative, outcomes.
➢ Among the 15 negative trials, 6 were unpublished and 2 others were misreported as positive. Seven other negative trials (47%) were reported transparently (as negative), an improvement over the low (11%) rate found earlier with the older antidepressants.
➢ Statistical comparison of the newer and older drug datasets indicated that transparent reporting had improved overall, mainly among negative trials. Yet compared to positive trials, the rate of transparent reporting for negative trials remains low.
➢ Using meta-analysis to compare drug efficacy based on FDA versus published data, we found less inflation of drug efficacy among newer, compared to older, antidepressants.

**What do these findings mean?:**

➢ Reporting bias persists but appears to have diminished for newer, compared to older, antidepressants.
➢ We do not know whether these results extend to drugs beyond the antidepressants studied here, nor do we know whether they extend to drug safety, as opposed to efficacy.
➢ Reporting bias remains a significant impediment to researchers and medical decision-makers, so further efforts are needed to improve transparent reporting in the scientific literature.

## Introduction

Reporting bias can lead to overestimates of efficacy and/or underestimates of harms and thus undermine the evidence base regarding drugs and other interventions. Reporting bias takes several forms, including study publication bias and outcome reporting bias [1]. With study publication bias, entire studies are published or not depending on their results; with outcome reporting bias, studies are published but their outcomes are reported selectively depending on their results.

One of the early studies on reporting bias in antidepressant trials was published by our group [2]. Examining 12 second-generation antidepressants approved by the Food and Drug Administration (FDA) between 1986 and 2004, we found strong evidence for both study publication bias and outcome reporting bias. Because drug companies must report results of all Phase II/III trials to the FDA in order to gain approval for a new drug, FDA review documents can be considered a gold standard, an unbiased sample of all studies undertaken. Compared to trial results in FDA review documents, results published in journals inflated the apparent efficacy of antidepressants over placebo both in terms of proportion of positive trials and effect size (ES).

Using similar methodology, evidence for reporting bias has also been found among drugs for the treatment of schizophrenia [3] and of anxiety disorders [4], although not to the extent observed in antidepressant trials. Nor is reporting bias limited to psychotropic drugs—it has been documented for both pharmacological and nonpharmacological interventions across medical indications [5–9], and it appears to exist in social, biological, and physical sciences, as well [10].

An examination of papers published in all disciplines between 1990 and 2007 suggested an increase in reporting bias over time [10]. Since then, however, there have been important transparency-related policy changes, such as requirements for registration of clinical trials in 2005 [11,12] and for reporting of the trial results mandated by the Food and Drug Administration Amendments Act (FDAAA) of 2007 [13], and recent work suggests that the level of transparency has improved [14,15].

This raises the question, has the level of transparency of clinical trials changed specifically for the drugs for which reporting bias has perhaps been best described, namely antidepressants? Since our 2008 publication, several new antidepressant drugs have entered the United States market. Using the earlier study of older antidepressants for comparison, the current study aims to similarly determine whether, and to what degree, the apparent efficacy of the newer drugs has been inflated in published journal articles. More specifically, it asks, does trial outcome (positive or not) still influence whether and how the trial is reported? And does reporting bias still inflate ES?

## Methods

### Data procurement

#### FDA-registered trials

This study extends the methodology of a previously published study of reporting bias, which examined 74 trials of 12 older antidepressants [2]. Using medical and statistical reviews within FDA drug approval packages (https://www.accessdata.fda.gov/scripts/cder/daf/) [16], we identified all Phase II or Phase III double-blind placebo-controlled acute monotherapy trials for major depressive disorder on 4 newer antidepressants (Table 1).

**Table 1:** List of antidepressants included in the older and newer cohorts of RCTs.

Drug group and cohort of RCTs	Approval date	Generic name	Brand name
Older [2]	December 1987	fluoxetine	Prozac
	December 1992	paroxetine	Paxil
	December 1993	venlafaxine	Effexor
	December 1994	nefazodone	Serzone
	June 1996	mirtazapine	Remeron
	October 1996	bupropion sustained release	Wellbutrin SR
	July 1997	sertraline	Zoloft
	October 1997	venlafaxine extended release	Effexor XR
	July 1998	citalopram	Celexa
	December 1999	paroxetine controlled release	Paxil CR
	August 2002	escitalopram	Lexapro
Newer	February 2008	desvenlafaxine	Pristiq
	January 2011	vilazodone	Viibryd
	July 2013	levomilnacipran	Fetzima
	September 2013	vortioxetine	Trintellix

RCT, randomized controlled trial.

### Literature search

Having identified the inception cohort of premarketing trials registered with the FDA, we used PubMed to search for matching publications reasonably discoverable by clinicians. Example search syntax for one drug was “desvenlafaxine[title] placebo (“major depressive disorder” OR “major depression”).” From the search output, titles and abstracts were screened to include journal articles focused on the overall efficacy of the drug in question for major depressive disorder; thus, we excluded articles focused on other indications, subsets with specific comorbid conditions, particular symptom clusters, safety (as opposed to efficacy), specific demographic samples, trials lacking a parallel design (add-on, open-label, crossover), trials that were not placebo controlled, trials not involving acute treatment (long-term trials, including maintenance trials), and trials involving other routes of administration. A literature search for the FDA-registered trials was also carried out independently by author YD in the context of a separate publication [17]. Separately, TF and YO identified the trials in ClinicalTrials.gov using the “Other Study ID Numbers” field and identified corresponding publications using the “Publication of Results” field. Matching of FDA-registered trials to publications was confirmed using trial design, duration, drugs used (study drug, placebo, active comparator), and number of participants randomized to each treatment arm. The preferred publication type was a stand-alone article, an article reporting on a single trial, with exceptions allowed as previously described [3].

### Confirmation of nonpublication

For desvenlafaxine and vilazodone, we were unable to identify publications corresponding to all of the FDA-registered trials, so following a method reported previously [3], we searched for bibliographic information on said trials within recent review articles [18–23], whose authors made use of additional databases, including EMBASE [18,22], ClinicalTrials.gov [22,23], and Cochrane Central [18]. Additionally, the authors of one article contacted the sponsor [22], and the authors of another article were employees of the sponsor [20].

### Data extraction

As with previous studies [2,3], we employed double data extraction and entry. Data were extracted and entered by 3 teams (ET with 3 assistants; YD; TF and YO), compared using Boolean formulas in Excel, and reconciled for any discrepancies. For each trial, we extracted the results on the primary outcome from the FDA reviews, including the summary statistics and the FDA reviewer’s judgment as to whether the trial was positive, i.e., whether it provided “substantial evidence of effectiveness” for purposes of marketing approval [24].

From the corresponding journal articles, consistent with our previous study of the apparent (to the average clinician-reader) efficacy of antidepressants [2],we extracted the summary statistics on the apparent primary outcome. This was defined as the drug–placebo comparison highlighted as the trial’s main result by virtue of its being reported first in the article’s results section.

### Data analysis

#### Descriptive examination of trial results

Many trials consisted of 2 or more treatment arms compared to a common placebo group, resulting in 2 or more *P* values. Treatment-arm-level *P* values reported by the FDA (P_FDA_) were compared to *P* values reported in corresponding journal articles (P_Journal_). Because of nonindependence (the same placebo group could be represented in 2 or more datapoints), they were examined descriptively in the form of scatterplots, one for each cohort of trials. The scatterplots necessarily excluded treatment arms whose results were not published (no P_Journal_ values).

#### Transparent publication

We considered a trial to be published transparently if the trial was published in a way that was consistent with the FDA report of that trial. Transparent publication was deemed absent when (a) the trial results were not published (study publication bias) or (b) the results were published but in a way that conflicted with the FDA report (outcome reporting bias). For example, if a trial was reported by the FDA to be negative (nonsignificant on the primary outcome), but the publication conveyed a positive overall result by emphasizing statistically significant results in the beginning of the results section (see above re apparent primary outcome) and the abstract, that trial was deemed not transparently published.

We examined 2 predictors of transparent publication. The first was trial cohort (i.e., older versus newer antidepressants). The second was trial outcome according to the FDA report—positive (study drug clearly statistically superior to placebo on the primary outcome) or not positive. The main model also included a third variable for the interaction between the first 2 predictors. To estimate the associations, within Stata 11 [25], we employed Firth (penalized) logistic regression using the module *firthlogit* [25–27]. As a secondary analysis, we employed exact logistic regression [26] using the module *exlogistic* [25]. (These methods were chosen because, in the context of rare events, such as FDA-positive trials that are not transparently published, standard logistic regression fails. Please see S1 Text for elaboration.) We also undertook the following post hoc univariable analyses: Because transparent publication is arguably more likely to occur with positive than negative trials, we examined the effect of cohort within each of these subsets; similarly, we examined the effect of trial outcome within each cohort.

#### Meta-analysis of effect sizes

The meta-analysis portion of this study is reported as per the Preferred Reporting Items for Systematic Reviews and Meta-Analyses (PRISMA) 2020 guideline (S1 Checklist).

We examined whether reporting bias misinformed the public by comparing one meta-analysis (MA) using trial data obtained from FDA reviews to a second MA using data from the corresponding publications. The MAs were conducted using the *metan* module in Stata 11 [25], with random effects pooling and the DerSimonian–Laird estimator for heterogeneity. The resulting effect measures (standardized mean difference Hedges’ g ± 95% confidence interval) obtained by author ET were verified against those obtained independently by author YD. As in previous work [2–4], for each multiple-dose trial, we used fixed effects MA to obtain a single trial-level ES; to avoid a spuriously low standard error, each trial’s shared placebo n was counted once rather than redundantly for each dose group.

We then compared the results of the MAs, with effect size inflation (ESI), presumably due to reporting bias, calculated as journal-based ES (ES_Journal_) minus FDA-based ES (ES_FDA_). To facilitate visual comparison of these values, we exported the Stata-generated forest plots to vector-based graphics software (Intaglio version 3.9), which allowed corresponding ES_FDA_ and ES_Journal_ values to be placed alongside one another. Such pairwise forest plots were generated showing ES values at the level of trial, drug, and cohort.

Because journal-based ES and FDA-based ES are not independent (both derived from the same set of trials), we did not perform a formal statistical comparison through, for instance, meta-regression. As an exploratory method, we did perform multivariate MA, which is capable of handling such dependency, but it is limited in another respect. As explained further in the Supporting information, multivariate MA relies on the correlation between paired FDA-based and journal-based ES values, and complete pairs exist for published trials but not for unpublished trials. Because unpublished trials, compared to published trials, are much more likely to be negative and have systematically smaller ES values [2], journal-based ES values are missing not at random. Thus, the multivariate approach is less well suited to the examination of study publication bias than to outcome reporting bias. Because our dataset contains both of these forms of reporting bias, results of the multivariate MA are provided as Supporting information (S1 Text and S8 Fig).

We included only doses approved by the FDA, as reflected in the Dosage and Administration section of the product label. While this wording in this section was clear in many cases, in others, it was ambiguous; thus, for certain doses, arguments could be made for both inclusion and exclusion. We resolved this by conducting a primary MA using broad dose inclusion criteria and a sensitivity MA using narrow or restrictive dose inclusion criteria. For rationale and elaboration, please see legend to **Table 2** and Table C in S1 Text. As explained in the latter, for dose reasons, one trial (vilazodone #244) was excluded from both MAs.

**Table 2:** FDA-registered trials and corresponding publications for cohort of 4 newer antidepressants.

Drug	Trial Number	Registry Identifier	Total N	Dose Groups[n]	Comparator [n]	Trial Outcome per FDA	Publication
Desvenlafaxine	223	*n/a*	213	200 mg^1^ [63], 400 mg^1^[72], pbo[78]	—	Negative	
	304	NCT00063206	234	’100 mg or 200 mg’ ^1^[120], pbo[114]	—	Negative	Liebowitz 2007 [47]
	306	NCT00072774	461	100 mg^1^[114], 200 mg^1^[116], 400^1^[113], pbo[118]	—	Positive	DeMartinis 2007 [48]
	308	*n/a*	369	200 mg^1^[121], 400^1^[124], pbo[124]	—	Positive	Septien-Velez 2007 [49]
	309	NCT00090649	364	’200 or 400 mg’ ^1^[110], pbo[120]	Venlafaxine “75 or 150 mg” [127]	Negative	Lieberman 2008 [27]
	317	NCT00087737	350	’200 or 400 mg’ ^1^[110], pbo[125]	Venlafaxine “150 or 225 mg” [115]	Negative	
	320	*n/a*	235	’200 or 400 mg’ ^1^[117], pbo[118]	—	Negative	Feiger 2009 [50]
	332	NCT00277823	447	50 mg^2^[150], 100 mg^1^[147], pbo[150]	—	Positive	Liebowitz 2008 [51]
	333	NCT00300378	483	50 mg^2^[164], 100 mg^1^[158], pbo[161]	—	Positive	Boyer 2008 [52]
Levomilnacipran	F02695 LP 202	EudraCT 2006-002404-3	553	75 mg-100 mg^2^[277],pbo[276]	__	Positive	Montgomery 2013 [53]
Levomilnacipran (cont’d)	LVM-MD-01	NCT00969709	713	40 mg^2^[178],80 mg^2^[179],120 mg^2^[180],pbo[176]	__	Positive	Asnis 2013 [54]
	LVM-MD-02	NCT00969150	357	40 mg-120 mg^2^[175],pbo[182]	__	Negative	Gommoll 2014 [55]
	LVM-MD-03	NCT01034462	434	40 mg-120 mg^2^[222],pbo[220]	__	Positive	Sambunaris 2014 [56]
	LVM-MD-10	NCT01377194	562	40 mg^2^[188],80 mg^2^[188],pbo[186]	__	Positive	Bakish 2014 [57]
Vilazodone	CLDA-07-DP-02	NCT00683592	468	40 mg^2^[235],pbo[233]	__	Positive	Khan 2011 [58]
	GNSC-04-DP-02	NCT00285376	409	40 mg^2^[205],pbo[204]	__	Positive	Rickels 2009 [59]
	244	*n/a*	289	20 mg-100 mg^0^[86], pbo[95]	Fluoxetine 20 mg [89]	Negative	Not published
	245	*n/a*	517	10–20 mg^1^[104],40–60 mg^1^[97],80–100 mg^0^[93],pbo[99]	Fluoxetine 20 mg [92]	Negative	Not published
	246	*n/a*	483	10 mg^0^[119],20 mg^1^[121],pbo[128]	Citalopram 20 mg [115]	Negative	Not published
	247	*n/a*	228	5 mg-20 mg^1^[113],pbo[115]	__	Negative	Not published
	248	*n/a*	533	20 mg^1^[132],10 mg^0^[133],5 mg^0^[140],pbo[128]	__	Negative	Not published
Vortioxetine	303	NCT00672958	597 (tx’d)	5 mg^1^[299],Pbo[298]	—	Negative	Jain 2013 [60]
	304	NCT00672620	611 (tx’d)	2.5 mg^0^[153], 5 mg^1^[153], pbo[153]	Duloxetine 60 mg [152]	Negative	Mahableshwarkar 2013 [61]
	305	NCT00735709	560	Pbo[140], 1 mg^0^[140], 5 mg^1^[140], 10 mg^1^[140]	—	Positive	Henigsberg 2012 [62]
	315	NCT01153009	591	15 mg^1^[145],20 mg^2^[147],pbo[153]	Duloxetine 60 mg [146]	Positive	Mahableshwarkar 2015(a) [63]
	316	NCT01163266	457	10 mg^1^[154],20 mg^2^[148],pbo[155]	—	Positive	Jacobsen 2015 [64]
	317	NCT01179516	434 (tx’d)	10 mg^1^[143],15 mg^1^[142],Pbo[149]	—	Negative	Mahableshwarkar 2015(b) [65]
	11492A	NCT00839423	425	5 mg^1^[108], 10 mg^1^[100], pbo[105]	Venlafaxine 225 mg [112]	Positive	Alvarez 2012 [66]
	11984A	NCT00635219	766 (tx’d)	2.5 mg^0^[155], 5 mg^1^[155], 10 mg^1^[151]	Duloxetine 60 mg [149]	Negative	Baldwin 2012 [67]
	13267A	NCT01140906	604	15 mg^1^[149],20 mg^2^[151],pbo[158]	Duloxetine 60 mg [146]	Positive	Boulenger 2014 [68]

Sample sizes (N/n) are number randomized unless specified as treated (“tx’d”). Superscripts in Dose Groups column correspond to dose inclusion levels explained in Table C in S1 Text.

^0^Dose group included in neither MA (excluded from both).

^1^Dose group included in primary MA.

^2^Dose group included in both primary and sensitivity MAs.

FDA, Food and Drug Administration; MA, meta-analysis.

## Results

The FDA-registered trials and their corresponding publications are listed in **Table 2**. For the cohort of 4 newer antidepressants, there were 30 applicable trials with 13,747 participants, while for the cohort of older antidepressants, there were 74 applicable trials with 12,564 participants. Median trial sample sizes for the newer and older cohorts were 439.5 and 147.5, respectively (Z = 6.72, *P* < 0.001 by Wilcoxon rank-sum test), different by a factor of 3.

### Results at level of treatment arm

**Fig 1:**
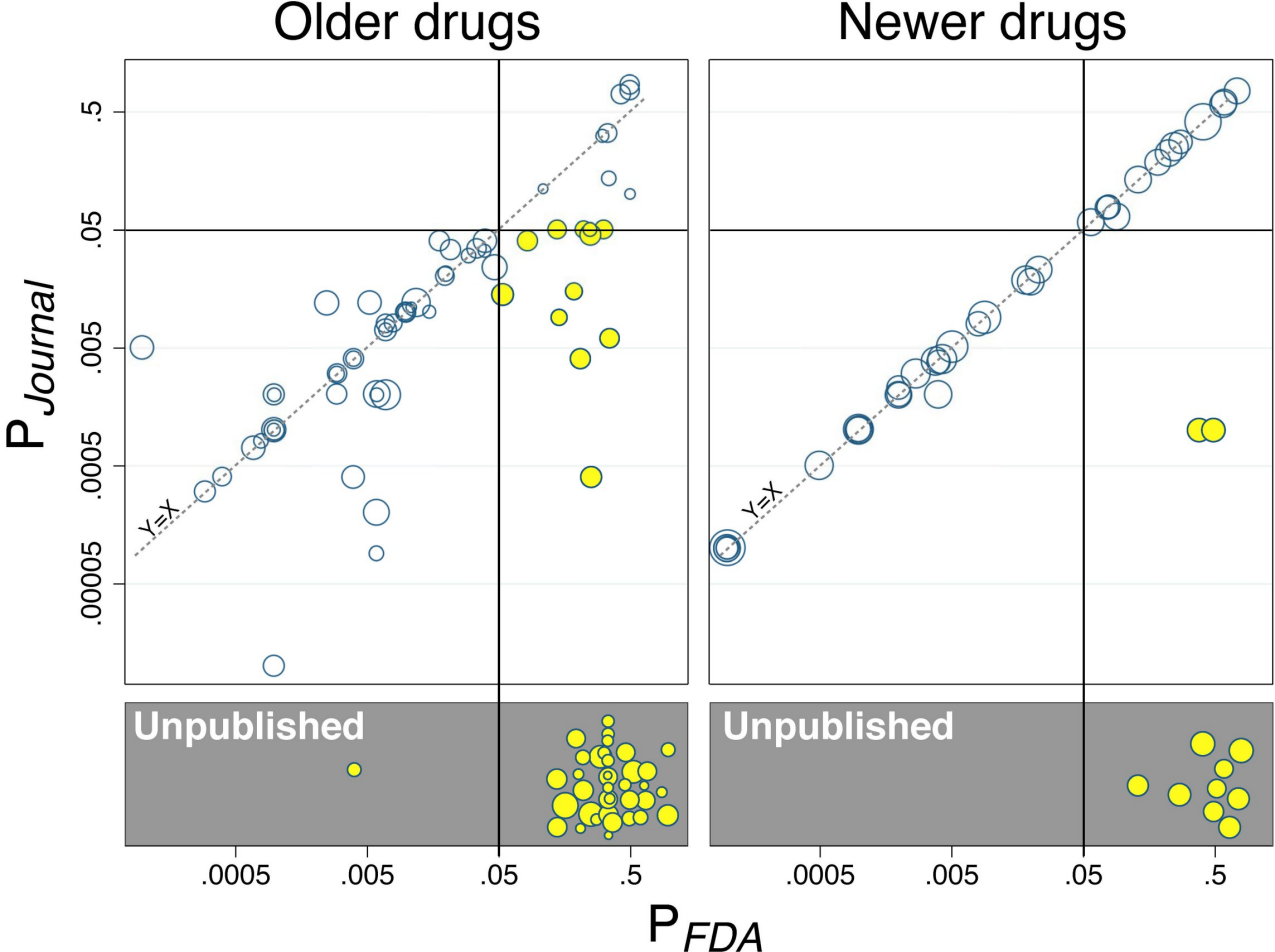
Reporting of *P* values in publications vs. FDA reviews for older and newer cohorts of antidepressant drugs. *P* values reported by the FDA (P_FDA_, horizontal axes) are compared to those in corresponding journal articles (P_Journal_, vertical axes). The older and newer antidepressants are shown in the left and right plots, respectively. Each data point represents a drug treatment arm compared to placebo, with area proportional to the sum of their sample sizes. Dashed diagonal represents concordance between P_FDA_ and P_Journal_, i.e., an absence of reporting bias. Cases of outcome reporting bias, where P_FDA_ is NS but P_Journal_ is reported as significant, are highlighted in yellow. (The 2 yellow circles far off the Y = X diagonal in the right-hand panel represent desvenlafaxine trials 309 and 317 (please see text).) Unpublished treatments arms, with P_FDA_ values but no corresponding P_Journal_ values, are shown in the gray boxes and highlighted in yellow. FDA, Food and Drug Administration; NS, not significant.

The total number of treatment arms was 149, with 101 and 48 in the older and newer cohorts, respectively. **Fig 1** plots the *P* values of all these arms against placebo as reported in the journals versus as confirmed in FDA reviews: It shows, among the 104 published treatment arms, a greater proportion of treatment arms lying along the Y = X (P_Journal_ = P_FDA_) diagonal in the newer cohort, i.e., greater concordance between journal- and FDA-based data. The proportion of unpublished treatment arms (gray boxes) was 36% (36/101, CI_95%_ 26% to 45%) for the older cohort versus 19% (9/48, CI_95%_ 8% to 30%) for the newer cohort.

### Transparent publication

For the 4 newer antidepressants, **Table 2** and S1 Fig show each trial’s overall outcome, as determined by the FDA, and its corresponding publication status. Of these 30 trials, the FDA deemed 15 (50%) to be positive, i.e., statistically significant on the prespecified primary outcome, consistent with the proportion previously reported for the older cohort [2]. Among these 15 FDA-positive trials, all were published in agreement with the FDA (transparently reported as positive). Among the 15 not-positive trials, 7 (47%) were transparently published (as nonsignificant), a higher proportion than that observed for the older cohort (4/37 = 11%) [2]. The remaining 8 (53%) FDA-negative trials in the newer cohort were not transparently published.

Six of these were simply not published. One desvenlafaxine trial was referred to in one review publication as “an unpublished report with the code name Des 223” [18]; in a second review publication authored by employees of the sponsor, it was referred as “data on file” [20]. Regarding vilazodone, one review publication referred to 5 trials (#244 to 248) as “astonishingly unfavorable” and cited the FDA drug approval package and no publications; a second review publication listed them in a paragraph and a data table devoted to unpublished trials.

Two other FDA-negative trials—desvenlafaxine EU trial 309 and US trial 317—were published but classified as not transparently published for 2 reasons. First, they were published solely in the form of a single positive “pooled analysis” paper [27]. (This form of reporting bias has been previously described in the antidepressant literature [28].) To be classified as transparently reported, the 2 trials should have been published in separate stand-alone papers highlighting their nonsignificant results, or published in a combined article highlighting the 2 nonsignificant results. Second, in the journal article, a nonprimary method of handling dropouts (MMRM instead of LOCF; Table D in S1 Text) was used, leading to statistically significant pooled results. (Although pooling trials increases statistical power, this alone would not have yielded a statistically significant result. Via post hoc MA of the FDA-reported primary results for these 2 trials, we calculated Hedges’ g = 0.10 (CI_95%_ −0.08 to 0.28, *P* = 0.27).) These significant results were highlighted in the abstract and beginning of the results section. Meanwhile, the nonsignificant results from the individual trials were reported beginning on the fifth page of the results section and not in the abstract.

**Fig 2:**
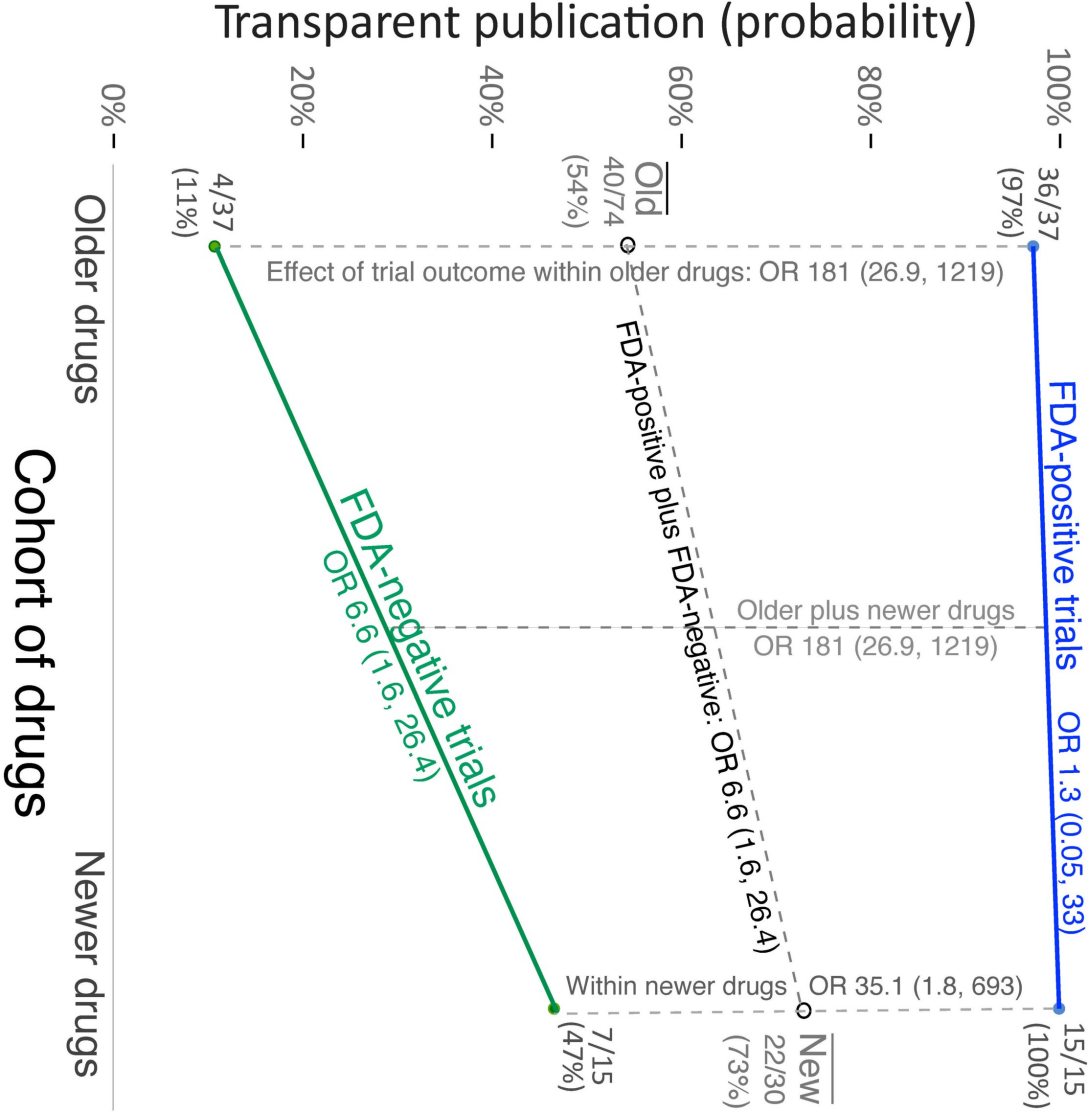
Interaction plot illustrating the effects of trial outcome and cohort (older vs. newer antidepressants) on transparent publication. For all trials regardless of outcome (dashed oblique line), the proportion of transparently reported trials increased from 54% (older drugs) to 73% (newer drugs). Within the subset of FDA-positive trials (blue line), transparent reporting, which was already nearly 100% for the older cohort, showed no further increase. By contrast, within FDA-negative trials (green line), transparent reporting increased from 11% to 47%. FDA, Food and Drug Administration.

The effects of trial outcome and cohort on transparent reporting were examined using logistic regression. Please refer to **Fig 2** for all counts and proportions, as well as odds ratios. (Further logistic regression results are available in Table A in S1 Text.) With respect to the variable for trial outcome, transparent reporting occurred more often for FDA-positive than for FDA-negative trials (OR 181, CI_95%_ 26.9 to 1,219, *P* < 0.001). Post hoc univariable analyses showed significant effects of trial outcome within the older cohort (OR 181 CI_95%_ 26.9 to 1,219, *P* < 0.001), consistent with findings reported earlier [2], and within the newer cohort (OR 35.1, CI_95%_ 1.8 to 693, *P* = 0.019). Within the newer cohort, 15 of 15 (100%) positive trials were reported transparently versus 7 of 15 (47%) negative trials.

With respect to the variable for cohort, the overall proportion of transparently reported trials increased from 54% to 73%. Controlling for trial outcome, trials in the newer cohort were 6.6 times more likely to be transparently reported than trials in the older cohort (OR 6.6, CI_95%_ 1.6 to 26.4, *P* = 0.008).

Post hoc analyses suggested that the higher rate of transparent publication in the newer cohort was limited to negative trials, which increased from 11% to 47%. Negative trials in the newer cohort were 6.6 times more likely to be transparently reported than negative trials in the older cohort (OR 6.6, CI_95%_ 1.6, 26.4, *P* = 0.008), equal to the abovementioned main effect of cohort. By contrast, positive trials were transparently reported approximately 100% of the time for both cohorts; thus, the post hoc univariable analysis showed no effect (OR 1.3, CI_95%_ 0.05 to 33, *P* = 0.88).

As shown in Table A in S1 Text, the multivariable model’s interaction effect was nonsignificant (OR 0.19, CI_95%_ 0.006 to 6.7, *P* = 0.36); omitting the interaction term had little impact on the multivariable models’ 2 main effects. For all of the abovementioned analyses, similar results were obtained using exact logistic regression (S2 Fig and Table A in S1 Text).

### Meta-analysis

Dose groups and trials included and excluded in the primary and sensitivity MAs are listed in Table C in S1 Text. Meta-analytic trial-level results from Stata, including forest plots, based on data from the FDA and the published literature, and for both primary and sensitivity MAs, are shown in S3–S6 Figs and Tables E-H in S1 Text.

S7 Fig is a forest plot comparing trial-level ES based on FDA versus journal data for each of the four newer antidepressants (using broad dose inclusion criteria). In S7 Fig, not-transparently published trials are highlighted for desvenlafaxine and vilazodone, which give rise to the observed (FDA- versus journal-based) ES differences at the level of drug (quantified below). For the other 2 drugs, levomilnacipran and vortioxetine, all trials were deemed transparently published (none highlighted otherwise); thus, their FDA- and journal-based ES values, at the level of both trial and drug, are virtually the same.

**Fig 3:**
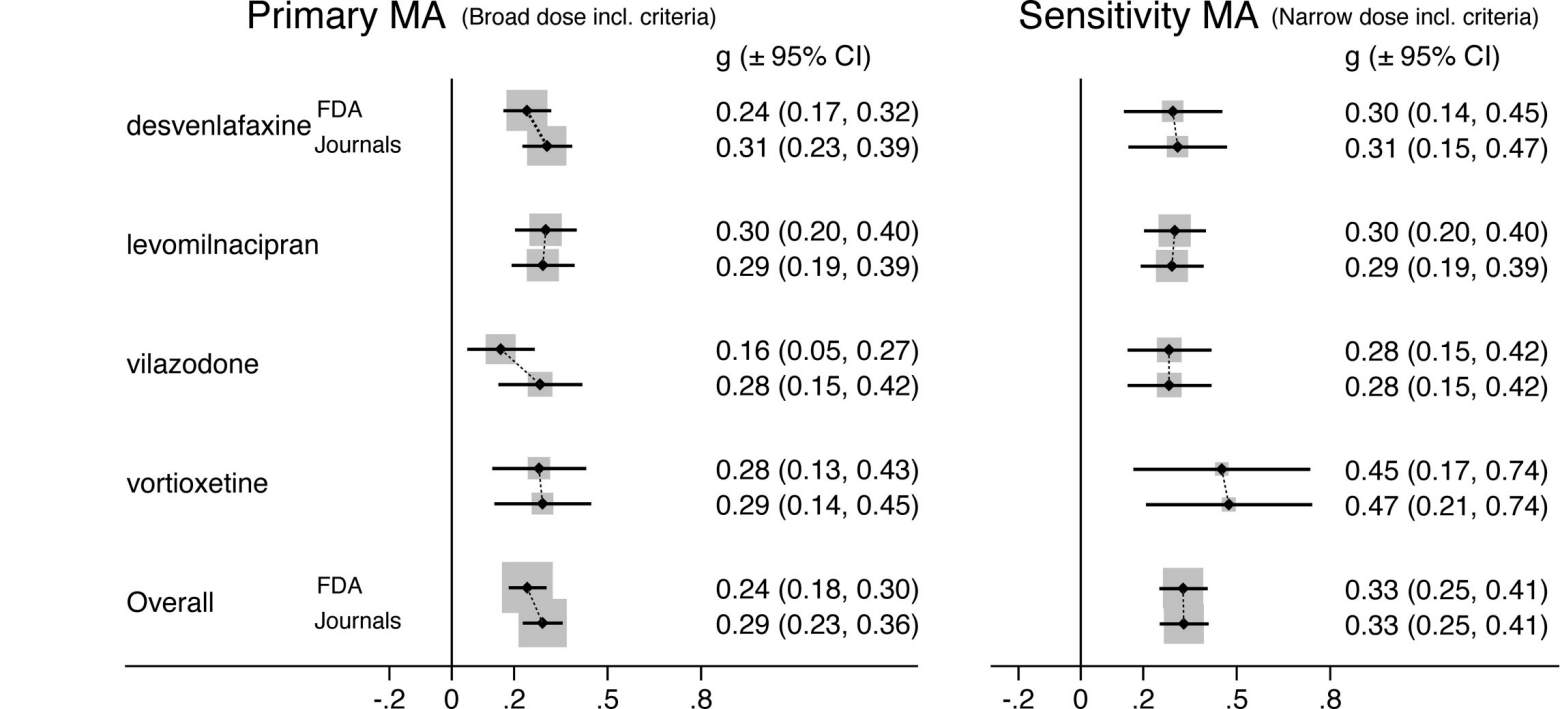
Meta-analytic ES of 4 newer antidepressants derived from trial reports in FDA reviews vs. journal articles. The primary (left panel) and sensitivity (right panel) MAs were based on broad and narrow/restrictive dose inclusion criteria, respectively, as described in text. For each antidepressant, 2 drug-level ES values are shown, one based on clinical trial data from FDA reviews and one based on data from the journal articles. ES, effect size; g, Hedges’s g; FDA, Food and Drug Administration; MA, meta-analysis.

The abovementioned drug-level ES values are summarized and compared in **Fig 3**. In the primary MA (left panel), as mentioned above, ESI was largest for vilazodone (0.28 − 0.16 = 0.12), followed by desvenlafaxine (0.31 − 0.24 = 0.07). The overall FDA-based Hedges’ g for the 4 newer antidepressants was 0.24 (CI_95%_ 0.18, 0.30), while the overall journal-based ES was 0.29 (CI_95%_ 0.23, 0.36), for an ESI of +0.05.

In the sensitivity MAs (right panel of **Fig 3**),which employed restrictive/narrow dose inclusion criteria (see Methods), ES values were generally higher, especially for the FDA-based values, bringing them into closer alignment with the journal-based values. Thus, the abovementioned ESIs for vilazodone and desvenlafaxine decreased to nearly zero. The overall FDA-based Hedges’ g in these analyses was 0.33 (CI_95%_: 0.25, 0.41), while the overall journal-based value was 0.33 (CI_95%_: 0.25, 0.41), resulting in an ESI of approximately zero (+0.0).

**Fig 4:**
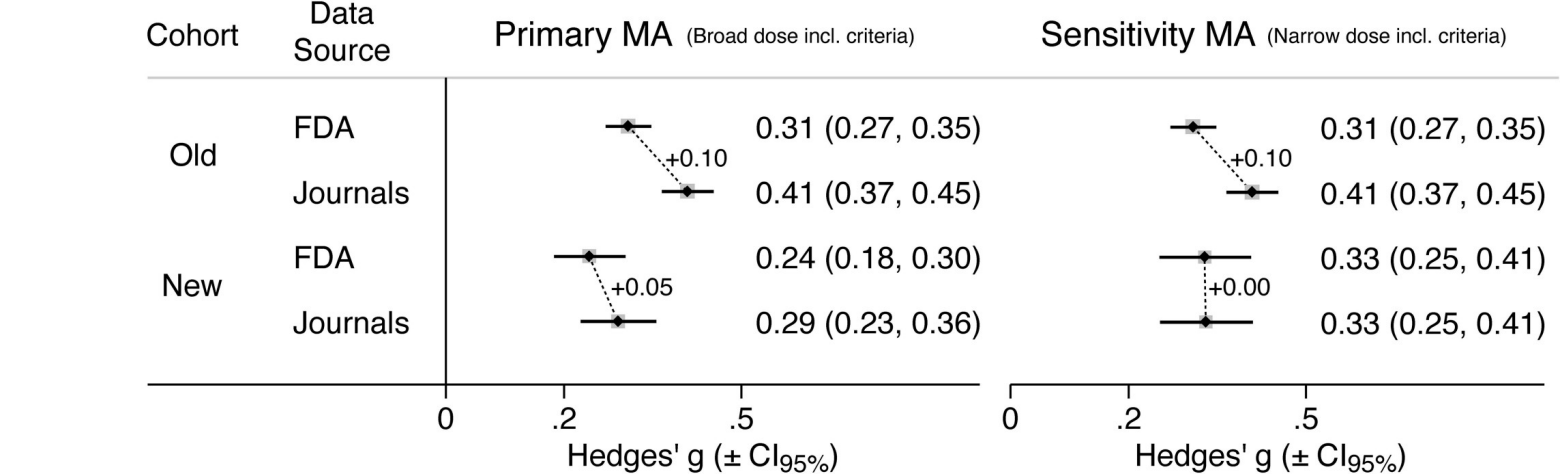
Comparison of overall ES, derived from FDA reviews vs. journal articles, for older and newer antidepressants. For the newer antidepressants, ESI was smaller than that previously reported for the older antidepressants. ES, effect size; ESI, effect size inflation; FDA, Food and Drug Administration; MA, meta-analysis.

Fig 4 compares the overall FDA- versus journal-based ES values for the newer versus the older cohort of antidepressants. As previously reported, the overall ESI for the older cohort was 0.10 (= 0.41 − 0.31), larger than the ESI found in the primary and sensitivity MA for the newer cohort. For additional context, ESI for individual drugs in the older cohort ranged from 0.03 (paroxetine controlled release) to 0.22 (mirtazapine), with a median of 0.10 [2].

## Discussion

The data presented here suggest that reporting bias in the published literature on antidepressant drugs is still an important issue. Even within the cohort of newer antidepressants, statistical significance still has an undue influence on whether and how these trials are reported. Consistent with earlier work, we found that positive trials are much more likely to be transparently reported than negative trials, whether one looks within the newer cohort or at both cohorts combined. However, we also found evidence for improvement in reporting bias: Antidepressants trials, especially those deemed negative by the FDA, are more likely to be published transparently than they were previously. Regarding the meta-analytic results, though not the subject of formal statistical analysis, smaller ESI values for the newer, compared to the older, cohort also could be consistent with a decrease in reporting bias.

### Comparison with previous findings

Our findings are consistent with at least 5 other recent studies: (1) A study [29] of Phase III randomized controlled trials (RCTs) in pediatric patients compared conference abstracts from 2008 to 2011 to subsequent publications and found evidence for “reduced but ongoing publication bias” as compared to a similar study from 15 years earlier [30]. (2) Another research group found evidence for improvements in some, though not all, measures of transparency (registration rates, results reporting, publication rates) for drugs approved in 2014, compared to drugs approved in 2012 [31]. (3) In an examination of trials of both pharmacological and nonpharmacological treatments for depression, the prevalence of proper registration and reporting was improved but still very low, despite the fact that registration and reporting had been mandatory for several years [32]. (4) Examining drugs approved for cardiovascular disease and diabetes mellitus [14], another group found a decrease in publication bias (as well as an increase in registration) among trials for drugs approved by the FDA after, compared to before, the FDAAA of 2007. (5) The same group applied similar methodology to drugs approved for several indications treated by neurologists and psychiatrists, as well as other indications (anesthesia, constipation, fibromyalgia, pain) [33]. The latter 2 studies were restricted to “pivotal” trials, a designation often assigned post hoc to trials with positive outcomes; by contrast, the current study covers all efficacy trials regardless of outcome, including so-called “failed trials” [34,35]. The current study differs from the 5 abovementioned studies in that it focuses on one drug class for one indication (major depressive disorder), thus enabling MA.

### Possible explanations

How might we explain this apparent increase in transparency? There have long been many incentives to engage in reporting bias [36]. In the past, there was little awareness within the research and clinical communities that the problem existed, and pharmaceutical companies (and others) could engage in reporting bias without fear of detection. Since then, however, there has been a cultural change, and what was once standard practice is no longer considered acceptable. Numerous policy changes have been implemented, summarized elsewhere [37]. ClinicalTrials.gov was launched in 2000, but registrations initially lagged. In 2004—the year the FDA approved duloxetine, the newest drug within the older cohort of antidepressants [2]—the International Committee of Journal Editors (ICMJE) announced that prospective registration would be a precondition for publication. The following year saw a 73% increase in the registration rate over a span of just 5 months [38]. In 2005, the WHO International Clinical Trials Registry Platform (http://apps.who.int/trialsearch/Default.aspx) was launched. In 2007, the FDAAA was enacted [13], which legally mandated public registration of applicable clinical trials and called for the augmentation of ClinicalTrials.gov with a basic results database; in 2010, FDAAA was clarified and expanded in scope to include all Phase II to IV drug and device trials, adverse events, and basic results [39].

It seems reasonable to conclude that these policy changes played a major role in bringing about the increase in transparency suggested by the current study and the others mentioned above. However, given the level of attention directed toward reporting bias with antidepressants, in the form of lawsuits [39], numerous key publications [2,40–43], and new incentives to increase transparency, for instance, the Good Pharma Scorecard [31], it is possible that substantial improvement would have occurred without these policy changes.

### Implications, theoretical and practical

However, we must caution that, while the proverbial glass of transparency is now half full, it also remains half empty. Nothing less than full transparency should be considered acceptable in the realm of healthcare. Greater awareness of reporting bias is needed among researchers and clinicians so that they do not naively accept published research findings at face value.

The abovementioned policy changes should not be celebrated until compliance with them improves. In the case of FDAAA, apparently due to a lack of political will, enforcement has been lax, leading to over $5 billion in accrued fines remaining uncollected (http://fdaaa.trialstracker.net). Additionally, many journals that ostensibly support the ICMJE policy of preregistration continue to publish a substantial number of unregistered or belatedly registered trials [32].

Perhaps what is needed most is to eliminate reporting bias at its root. FDA reviews include the results of negative, as well as positive, studies, because the Agency receives study protocols before studies are undertaken, thus preventing drug companies from hiding the existence of studies or switching their outcomes. Although trial registries are intended to serve a similar purpose, they are separate from journals, in which the strength and direction of study results can continue to dictate submission and acceptance decisions. However, in an emerging peer review model known as Registered Reports [44], manuscripts are submitted and reviewed before studies are undertaken, leading to preliminary publication decisions based solely on the scientific question and methodological rigor. Registered Reports has been adopted, or offered as an option, by >300 journals in various fields (https://cos.io/rr/?_ga=2.192240618.1714708995.1570198509-367521697.1570198509, accessed November 20, 2021), but uptake among major medical journals has unfortunately lagged.

### Limitations and suggestions for future research

Despite the above-referenced consistency with previous findings, this study has several limitations that may affect generalizability. The scope of this study is limited to drug efficacy, so future research could examine whether transparency has also improved with regard to safety issues. For instance, we previously found that reporting of serious adverse events in the older antidepressant trials is often incomplete and inconsistent with FDA information [45]. Another scope-related limitation is that, because this study is restricted to antidepressants, these findings may not extend to other drug classes, which likely vary in signal-to-noise ratio (efficacy), proportion of “negative” trials, “need” for reporting bias, and hence potential for increased transparency. Further, this report pertains to Phase II/III premarketing trials only; the extent of reporting biases for Phase IV postmarketing trials is yet to be examined.

The most recently approved drugs in our sample were approved by the FDA in 2013, making it difficult to draw inferences about more recent changes in transparency practices. On the other hand, this study is a follow-up to our earlier review, making the contrast before versus after 2008 the topic of research. In addition to 2008 being the year of approval for the newest antidepressant in the older cohort, it was the year FDAAA was enacted, a milestone event for addressing reporting bias [13]. In any case, it was not possible to include drugs approved after 2013. The sample of older drugs was limited to those FDA-approved as monotherapy for major depressive disorder, and newer such drugs do not exist. More recent FDA approvals exist only for drugs FDA-approved as adjunctive therapy and/or for other depression-related indications such as treatment-resistant depression and postpartum depression, and our findings may not extend to reporting practices on such drugs.

For the newer, compared to the older, cohort, although the sample size was larger in terms of overall number of patients, it was smaller in terms of the number of drugs (4 versus 12, respectively) and trials (30 versus 74). However, as noted above, the drugs and trials in our sample were dictated by the drug development/approval process. Additionally, selective reporting practices may be correlated among trials for the same treatment from the same sponsor. (This limitation also applies to our earlier study [2]; among the 12 older antidepressants, one company sponsored 3 drugs and 3 companies sponsored 2 each.)

There are also limitations with respect to the statistical approach. First, the dichotomization of drugs into older and newer cohorts is somewhat arbitrary in that it is based on the timing of the earlier publication on the older cohort [2]. Second, because pharmaceutical companies often have publication strategies [46], within any given company, decisions whether to transparently publish its trials likely do not occur independently of one another. Third, the logistic regression model’s interaction term was nonsignificant; however, due to a limited sample size, and because transparent reporting of positive trials in the older cohort was already approximately 100%, leaving no room for improvement, the model was underpowered to detect such an effect. Fourth, the nonindependence of journal-based and FDA-based ES precluded formally contrasting them via meta-regression, so we were unable to determine whether the observed ESI is statistically significant. Fifth and relatedly, in contrasting the proportion of unpublished treatment arms between the older and newer cohorts, because many studies compared 2 or more dose arms to a single placebo group, the assumption of independence was not fully met.

In the current study, we found no change in the (high) level of transparent publication within FDA-positive trials. This is unsurprising—regardless of cohort, it is hard to conceive of a positive trial going unreported or being reported as negative. Hence, it could be argued that positive trials are uninformative and may hamper reporting bias signal detection. Therefore, in future analyses of reporting bias datasets, researchers may wish to consider focusing primarily on (more informative) negative trials.

In the sensitivity MA, compared to the primary MA, the gap between ES_Journals_ and ES_FDA_ was diminished. The sensitivity MA employed narrow dose inclusion criteria, i.e., was limited to those doses unambiguously approved by the FDA. The FDA determines effective dose ranges based on how “successful” the various doses are in Phase II/III trials. In the narrowly defined dose range, the sample was limited to clearly successful (FDA-positive) data, which are associated with larger ES values and higher levels of transparent publication (less reporting bias).

## Conclusions

In this study, we found a persistence of reporting bias within a cohort of newer antidepressants approved since 2008. However, compared to the cohort of older antidepressants, reporting bias in the newer cohort appeared to decrease, and neither study publication nor outcome reporting bias was found among trials for the 2 newest antidepressants (both approved in 2013). The observed improvement in transparency may be due to cultural and policy changes over the past decade. Further efforts and vigilance are needed to maintain and build upon these improvements.

## Supporting information

S1 Fig Overall trial outcome according to FDA and publication status for the 4 newer antidepressants. Among 15 FDA-positive trials, all were published in agreement with the FDA (transparently reported as positive). Among 15 not-positive trials, 7 (47%) were transparently published (as nonsignificant), a higher proportion than that observed for the older antidepressants. FDA, Food and Drug Administration.
(EPS)

S2 Fig Stata commands underlying results displayed in similar figure in main paper. Above each line, the command for the primary method, penalized (Firth) logistic regression, is shown. Below each line, the command for the secondary method, exact logistic regression, is shown.
(EPS)

S3 Fig Forest plot based on data from FDA reviews using broad dose inclusion criteria. ES, effect size; FDA, Food and Drug Administration.
(EPS)

S4 Fig Forest plot based on data from journal articles using broad dose inclusion criteria. ES, effect size.
(EPS)

S5 Fig Forest plot based on data from FDA reviews using narrow dose inclusion criteria. ES, effect size; FDA, Food and Drug Administration.
(EPS)

S6 Fig Forest plot based on data from journal articles using narrow dose inclusion criteria. ES, effect size.
(EPS)

S7 Fig Forest plot comparing trial-level ES based on FDA vs. journal data for the 4 newer antidepressants (broad dose inclusion criteria). Red boxes indicate trials that were not transparently published. ES, effect size; FDA, Food and Drug Administration.
(EPS)

S8 Fig Graphical depiction of results from the multivariate MA. Blue asterisks and black plus signs represent trials of older and newer antidepressants, respectively. Blue and black ellipses indicate the 95% confidence regions for their respective summary effects. FDA, Food and Drug Administration; MA, meta-analysis; SMD, standardized mean difference.
(EPS)

S1 Text Table A. Results from penalized (Firth) logistic regression models. Effect of trial outcome and cohort on transparent reporting of trial outcome. Table B. Results from exact logistic regression models. Effect of trial outcome and cohort on transparent reporting of trial outcome. Table C. Rationale for inclusion of dosage groups in primary vs. sensitivity MA. Table D. Primary outcomes according to FDA review and journal articles. Table E. Numerical results (FDA-based MA; broad dose inclusion criteria). Table F. Numerical results (journal-based MA; broad dose inclusion criteria). Table G. Numerical results (FDA-based MA; narrow dose inclusion criteria). Table H. Numerical results (journal-based MA; narrow dose inclusion criteria). Table I. Results of the multivariate MAs. FDA, Food and Drug Administration; MA, meta-analysis
(DOCX)

S1 Checklist PRISMA 2020 Checklist. (DOCX)

S2 Checklist PRISMA for Abstracts Checklist. (DOCX)

S1 Data Logistic regression data in Stata (.dta) format. (DTA)

S2 Data Logistic regression data in Excel (.xlsx) format. (XLSX)

S3 Data Data used for FDA-based MA, BROAD dose inclusion criteria, Stata (.dta) format. FDA, Food and Drug Administration; MA, meta-analysis.
(DTA)

S4 Data Data used for JOURNAL-based MA, BROAD dose inclusion criteria, Stata (.dta) format. MA, meta-analysis.
(DTA)

S5 Data Data used for FDA-based MA, NARROW dose inclusion criteria, Stata (.dta) format. FDA, Food and Drug Administration; MA, meta-analysis.
(DTA)

S6 Data Data used for JOURNAL-based MA, NARROW dose inclusion criteria, Stata (.dta) format. MA, meta-analysis.
(DTA)

S7 Data Data used for FDA- and JOURNAL-based MAs, BROAD and NARROW dose inclusion criteria, Excel (.xlsx) format. FDA, Food and Drug Administration; MA, meta-analysis.
(XLSX)

## Abbreviations

ES: effect size
ESI: effect size inflation
FDA: Food and Drug Administration
FDAAA: Food and Drug Administration Amendments Act
ICMJE: International Committee of Journal Editors
MA: meta-analysis
RCT: randomized controlled trial
