# Supplementary material for: Selective publication of antidepressant trials and its influence on apparent efficacy: Updated comparisons and meta-analyses of newer versus older trials
Source: PLoS Med. 2022 Jan 19;19(1):e1003886. doi: 10.1371/journal.pmed.1003886 (PMC8769343; doi:10.1371/journal.pmed.1003886)
Supplement: S1 Text — Table A. Results from penalized (Firth) logistic regression models. Effect of trial outcome and cohort on transparent reporting of trial outcome. Table B. Results from exact logistic regression models. Effect of trial outcome and cohort on transparent reporting of trial outcome. Table C. Rationale for inclusion of dosage groups in primary vs. sensitivity MA. Table D. Primary outcomes according to FDA review and journal articles. Table E. Numerical results (FDA-based MA; broad dose inclusion criteria). Table F. Numerical results (journal-based MA; broad dose inclusion criteria). Table G. Numerical results (FDA-based MA; narrow dose inclusion criteria). Table H. Numerical results (journal-based MA; narrow dose inclusion criteria). Table I. Results of the multivariate MAs. FDA, Food and Drug Administration; MA, meta-analysis (DOCX) [file pmed.1003886.s009.docx]

# S1 Text. Supplementary Methods and Results

## Search syntax used for the four newer antidepressants:

desvenlafaxine[title] placebo ("major depressive disorder" OR "major depression

vilazodone[title] placebo ("major depressive disorder" OR "major depression

levomilnacipran[title] placebo ("major depressive disorder" OR "major depression

vortioxetine[title] placebo ("major depressive disorder" OR "major depression

## Additional background on logistic regression methods

Logistic regression was used because, in contrast to Fisher Exact test, it allows one to test the dependent variable against more than one independent variable simultaneously. However, standard logistic regression fails in the context of rare events, such as failure to transparently publish when trial results are FDA-positive, as it leads to biased maximum likelihood estimates [1], also known as “the problem of separation” [2]. To clarify, “The problem is not specifically the rarity of events, but rather the possibility of a small number of cases on the rarer of the two outcomes [3] ." (In our dataset, upon attempting standard logistic regression, Stata dropped all observations in which the trial results were FDA-positive and issued the error message “FDA = 0 predicts success perfectly?”)

One solution to this problem is to use exact logistic regression [4], which can be accomplished Stata using the exlogistic module within Stata. However, exact logistic regression is computationally intensive: “In samples with n > 200 and/or in cases with ‘many’ covariates and/or non-discrete covariates exact logistic regression will blow up working memory [1]."

A second variant of logistic regression, one not prone to this limitation, is penalized maximum likelihood estimation (PMLE) logistic regression, aka the Firth logistic regression [1,3,5] , which can be conducted using the Firthlogit module within Stata. Because of the possibility of future studies of this type with larger sample sizes, we employed Firth logistic regression as our primary method and exact logistic regression as the secondary method.

## Supplemental methods and results from logistic regression

For the logistic regression, supplemental data files are provided in both Stata (.dta) and Excel (.xlsx) formats. Below is a modified version of Figure 2 of the main paper showing the Stata commands used to produce the results shown in that figure.

Results from the primary method, Firth logistic regression, are presented below in **S1 Table**. However, while the univariable analyses in the main paper examine within subsets, the those in the tables below do not. For example, for the effect of cohort, the results below were generated using the Stata command "firthlogit tparent cohort, or", omitting the additional syntax shown in the figure above, namely "if pos_neg==1" (within FDA-positive trials) or "if pos_neg==0" (within FDA-negative trials). Similarly, for the effect of trial outcome, the results below were generated using the Stata command "firthlogit tparent pos_neg, or", omitting the additional syntax shown in the figure above, namely “in 1/74” (within older cohort) and the “in 75/104” (within newer cohorts), thus including both cohorts of antidepressants.

**Table A in S1 Text. Results from penalized (Firth) logistic regression models. Effect of trial outcome and cohort on transparent reporting of trial outcome**

| **Predictor variable** | **Univariable** | | **Multivariable** | | | |
| --- | --- | --- | --- | --- | --- | --- |
|  |  |  | **With interaction term** | | **Without interaction term** | |
|  | **OR (95% CI)** | **P value** | **OR (95% CI)** | **P value** | **OR (95% CI)** | **P value** |
| **Trial outcome per FDA**  Negative  Positive | 1  124 (22, 713) | <.001 | 1  181 (27, 1219) | <.001 | 1  192 (29, 1255) | <.001 |
| **Cohort of drug trials**  Old  New | 1  2.3 (0.9, 5.6) | 0.08 | 1  6.6 (1.6, 26.4) | 0.008 | 1  6.7 (1.7, 26.6) | 0.007 |
| **Trial outcome x Cohort** | –– | –– | 0.2 (.006, 6.7) | 0.364 | –– | –– |

Similar results were obtained using the secondary method, exact logistic regression, and are presented below (Table B in S1 Text). The Stata command used to generate the results shown in the top left cells of the table was "exlogistic tparent pos_neg, nolog"; for the cells below that, the command was "exlogistic tparent cohort, nolog". (In exlogistic commands such as these, ", nolog" can be omitted; it is an option that suppresses output of "sample space enumerations", which can be lengthy.)

**Table B in S1 Text. Results from exact logistic regression models. Effect of trial outcome and cohort on transparent reporting of trial outcome**

| **Predictor variable** | **Univariable** | | **Multivariable** | | | |
| --- | --- | --- | --- | --- | --- | --- |
|  |  |  | **With interaction term** | | **Without interaction term** | |
|  | **OR (95% CI)** | **P value** | **OR (95% CI)** | **P value** | **OR (95% CI)** | **P value** |
| **Trial outcome per FDA**  Negative  Positive | 1  176 (25, 7830) | <.001 | 1  247 (29, 12384) | <.001 | 1  265 (33, 12978) | <.001 |
| **Cohort of drug trials**  Old  New | 1  2.3 (0.9, 6.8) | 0.11 | 1  6.9 (1.4, 41) | 0.016 | 1  7.1 (1.5, 41) | 0.012 |
| **Trial outcome x Cohort** | –– | –– | 0.05 (.001, +Inf) | 1.00 | –– | –– |

## **Detailed meta-analytic results**

### **Dose inclusion criteria for meta-analysis**

For purposes of meta-analysis (MA), we aimed to include only doses approved by the FDA, as reflected in the Dosage and Administration section of the product label. While this wording was clear in many cases, in others it was ambiguous, i.e., arguments could be made for both including and excluding the dose in question (S3 Table, Inclusion Level 1, yellow shading). We addressed this by conducting two meta-analyses. The primary MA employed broad dose inclusion criteria, excluding only those dose groups that were clearly excluded (Inclusion Level 0, S3 Table, red shading). The sensitivity MA employed narrow dose inclusion criteria, restricted to dose groups that were clearly included (Inclusion Level 2, green shading). One trial, vilazodone #244, consisted of a single dose group deemed Inclusion Level 0, so it was excluded from both meta-analyses.

Table C in S1 Text. Rationale for inclusion of dosage groups in primary vs. sensitivity meta-analysis

| **Drug** | **Trial Number** | **Dosage Group** | **Inclusion Level** | **Rationale for Inclusion Level** |
| --- | --- | --- | --- | --- |
| Desvenlafaxine | 223 | 200 | 1 | Labeling: "In clinical studies, doses of 50 mg to 400 mg per day were shown to be **effective**, although no additional benefit was demonstrated at doses greater than 50 mg per day and adverse reactions and discontinuations were more frequent at higher doses."  However, while labeling calls these doses "effective", it reserves the words "recommended" and "therapeutic" for the 50-mg dose (see below). |
|  |  | 400 | 1 |  |
|  | 304 | '100 or 200' | 1 |  |
|  | 306 | 100 | 1 |  |
|  |  | 200 | 1 |  |
|  |  | 400 | 1 |  |
|  | 308 | 200 | 1 |  |
|  |  | 400 | 1 |  |
|  | 309 | '200 or 400' | 1 |  |
|  | 317 | '200 or 400' | 1 |  |
|  | 320 | '200 or 400' | 1 |  |
|  | 332 | 50 | 2 | Labeling: "The **recommended** dose for PRISTIQ is 50 mg once daily…The 50 mg dose is both a starting dose and the **therapeutic** dose." |
|  |  | 100 | 1 | "Effective" but greater than "recommended" 50-mg dose (see above). |
|  | 333 | 50 | 2 | "Recommended" and "therapeutic" dose. |
|  |  | 100 | 1 | "Effective" but greater than "recommended" 50-mg dose (see above). |
| Levomilnacipran | F02695 LP 202 | 75-100 | 2 | Product labeling, Dosage and Administration section:  "The recommended dose range for FETZIMA is 40 mg to 120 mg once daily, with or without food."  All doses used in these clinical trials fell within this range. |
|  | LVM-MD-01 | 40 | 2 |  |
|  |  | 80 | 2 |  |
|  |  | 120 | 2 |  |
|  | LVM-MD-02 | 40-120 | 2 |  |
|  | LVM-MD-03 | 40-120 | 2 |  |
|  | LVM-MD-10 | 40 | 2 |  |
|  |  | 80 | 2 |  |

| **Drug** | **Trial Number** | **Dosage Group** | **Inclusion Level** | **Rationale for Inclusion Level** |
| --- | --- | --- | --- | --- |
| Vilazodone | 244 | 20-100 | 0 | FDA medical review, p.25 of 259: "(52/66, 78%) patients received 80-100 mg/day." Thus, majority of patients in this trial received a dose that exceeded what was approved. (This trial was excluded from both main and sensitivity meta-analyses.) |
|  | 245 | 10-20 | 1 | (1) See comment attached to Study 246.  (2) No information available to ascertain what percentage of patients received approved dose (20mg). |
|  |  | 40-60 | 1 | FDA review of this study states that patients "Received 10 mg on Day 1, 20 mg/day on Days 2 and 3, 40 mg/day for Days 4- 14; if no clinical improvement was seen at Day 14, the dose was to be increased to 60 mg/day on Day 15." There is no information on the actual doses used. |
|  |  | 80-100 | 0 | Clearly exceeds highest approved dose (40 mg). |
|  | 246 | 10 | 0 | Clearly less than lowest (currently) approved dose (20 mg). |
|  |  | 20 | 1 | The 20-mg dose was not recommended at the time of approval (see CLDA-07-DP-02). Four years later (2015), labeling was liberalized to: "The recommended target dosage for VIIBRYD is 20 mg to 40 mg orally once daily." |
|  | 247 | 5-20 | 1 | This was a flexible dose study, in which dose increases to 10 followed by 20 occurred "at the discretion of the investigator, based on clinical response and tolerability." (Page 33 of 259.) No information in FDA review as to what the actual doses were, and there was no trial publication to consult. |
|  | 248 | 5 | 0 | Clearly less than lowest (currently) approved dose (20 mg). |
|  |  | 10 | 0 | Clearly less than lowest (currently) approved dose (20 mg). |
|  |  | 20 | 1 | Please see comments re study #246 (above) and CLDA-07-DP-02 (below). |
|  | CLDA-07-DP-02 | 40 | 2 | Labeling at time of Jan 2011 approval: "The recommended dose for VIIBRYD is 40 mg once daily." (see study #246 re later approval of 20-mg dose.) |
|  | GNSC-04-DP-02 | 20-40 | 2 | Per FDA medical review, p.46 of 249, only 13 of the 152 vilazodone-treated patients (8.6%) took 20 mg. Thus >90% took 40mg (see immediately above). |

| **Drug** | **Trial Number** | **Dosage Group** | **Inclusion Level** | **Rationale for Inclusion Level** |
| --- | --- | --- | --- | --- |
| Vortioxetine | 303 | 5 | 1 | Labeling recommends 20 mg as target dose (see below) but also states, "A dose decrease down to 5 mg/day may be considered for patients who do not tolerate higher doses". |
|  | 304 | 2.5 | 0 | Clearly less than lowest dose recommended in labeling. |
|  |  | 5 | 1 | See comment above re 5-mg dose. |
|  | 305 | 1 | 0 | Clearly less than lowest dose recommended in labeling. |
|  |  | 5 | 1 | See comment above re 5-mg dose. |
|  |  | 10 | 1 | Labeling recommends starting at 10mg then increasing to 20mg. |
|  | 315 | 15 | 1 | Labeling recommends starting at 10mg then increasing to 20mg. |
|  |  | 20 | 2 | Dose recommended unambiguously in labeling. |
|  | 316 | 10 | 1 | See comment above re 10-mg dose. |
|  |  | 20 | 2 | Dose recommended unambiguously in labeling. |
|  | 317 | 10 | 1 | See comment above re 10-mg dose. |
|  |  | 15 | 1 | See comment above re 15-mg dose. |
|  | 11492A | 5 | 1 | See comment above re 5-mg dose. |
|  |  | 10 | 1 | See comment above re 10-mg dose. |
|  | 11984A | 2.5 | 0 | Clearly less than lowest dose recommended in labeling. |
|  |  | 5 | 1 | See comment above re 5-mg dose. |
|  |  | 10 | 1 | See comment above re 10-mg dose. |
|  | 13267A | 15 | 1 | See comment above re 15-mg dose. |
|  |  | 20 | 2 | Dose recommended unambiguously in labeling. |

**Inclusion level refers to number of meta-analyses in which the dosage group in question was included.**

**Inclusion level 0 (red shading): Dose clearly excluded per labeling, so excluded from both main and sensitivity MAs.**

**Inclusion level 1 (yellow shading): Labeling ambiguously worded, with dose neither clearly included or clearly excluded, so dosage group included in main meta-analysis using broad dose inclusion criteria but not in sensitivity MA.**

**Inclusion level 2 (green shading): Dose clearly included per labeling, so dosage group included in both main and sensitivity MAs.**

**Table D in S1 Text. Primary outcomes according to FDA review and journal articles.**

| **Drug** | **Trial Number** | **FDA** | | **Journal article** | | |
| --- | --- | --- | --- | --- | --- | --- |
|  |  | **Primary outcome** | **Analytic strategy for handling dropouts** | **Primary outcome** | **Analytic strategy for handling dropouts** | **Reference** |
| Desvenlafaxine | 223 | Change from baseline to week 8 in HAMD-17 total score | LOCF |  |  |  |
|  | 304 | Change from baseline to week 8 in HAMD-17 total score | LOCF | Change from baseline to week 8 in HAMD-17 total score | LOCF | Liebowitz 2007 [6] |
|  | 306 | Change from baseline to week 8 in HAMD-17 total score | LOCF | Change from baseline to week 8 in HAMD-17 total score | LOCF | DeMartinis 2007 [7] |
|  | 308 | Change from baseline to week 8 in HAMD-17 total score | LOCF | Change from baseline to week 8 in HAMD-17 total score | LOCF | Septien-Velez 2007 [8] |
|  | 309 | Change from baseline to week 8 in HAMD-17 total score | LOCF | Change from baseline to week 8 in HAMD-17 total score | MMRM | Lieberman 2008 [9] |
|  | 317 | Change from baseline to week 8 in HAMD-17 total score | LOCF |  |  |  |
|  | 320 | Change from baseline to week 8 in HAMD-17 total score | LOCF | Change from baseline to week 8 in HAMD-17 total score | LOCF | Feiger 2009 [10] |
|  | 332 | Change from baseline to week 8 in HAMD-17 total score | LOCF | Change from baseline to week 8 in HAMD-17 total score | LOCF | Liebowitz 2008 [11] |
|  | 333 | Change from baseline to week 8 in HAMD-17 total score | LOCF | Change from baseline to week 8 in HAMD-17 total score | LOCF | Boyer 2008 [12] |
| Levomilnacipran | F02695 LP 202 | Change from baseline to week 10 on MADRS | MMRM | Change from baseline to week 10 on MADRS | MMRM | Montgomery 2013 [13] |
|  | LVM-MD-01 | Change from baseline to week 8 on MADRS | MMRM | Change from baseline to week 8 on MADRS | MMRM | Asnis 2013 [14] |
|  | LVM-MD-02 | Change from baseline to week 8 on MADRS | Not mentioned, probably MMRM | Change from baseline to week 8 on MADRS | MMRM | Gommoll 2014 [15] |
|  | LVM-MD-03 | Change from baseline to week 8 on MADRS | MMRM | Change from baseline to week 8 on MADRS | MMRM | Sambunaris 2014 [16] |
|  | LVM-MD-10 | Change from baseline to week 8 on MADRS | MMRM | Change from baseline to week 8 on MADRS | MMRM | Bakish 2014 [17] |
| Vilazodone | CLDA-07-DP-02 | Change from baseline to week 8 on MADRS | LOCF | Change from baseline to week 8 on MADRS | LOCF | Khan 2011 [18] |
|  | GNSC-04-DP-02 | Change from baseline to week 8 on MADRS | LOCF | Change from baseline to week 8 on MADRS | LOCF | Rickels 2009 [19] |
|  | 244 | Change from baseline to week 8 on HAMD-17 | LOCF |  |  |  |
|  | 245 | Change from baseline to week 8 on HAMD-17 | LOCF |  |  |  |
|  | 246 | Change from baseline to week 8 on HAMD-17 | Probably LOCF |  |  |  |
|  | 247 | Change from baseline to week 8 on HAMD-17 | LOCF |  |  |  |
|  | 248 | Change from baseline to week 8 on HAMD-17 | Probably LOCF |  |  |  |
| Vortioxetine | 303 | Change from baseline to 6 weeks on HAMD-24 | LOCF | Change from baseline to 6 weeks on HAMD-24 | LOCF | Jain 2013 [20] |
|  | 304 | Change from baseline to 8 weeks on HAMD-24 | LOCF | Change from baseline to 8 weeks on HAMD-24 | LOCF | Mahableshwarkar 2013 [21] |
|  | 305 | Change from baseline to 8 weeks on HAMD-24 | MMRM | Change from baseline to 8 weeks on HAMD-24 | MMRM | Henigsberg 2012 [22] |
|  | 315 | Change from baseline to week 8 on MADRS | MMRM | Change from baseline to week 8 on MADRS | MMRM | Mahableshwarkar 2015(a) [23] |
|  | 316 | Change from baseline to week 8 on MADRS | MMRM | Change from baseline to week 8 on MADRS | MMRM | Jacobsen 2015 [24] |
|  | 317 | Change from baseline to week 8 on MADRS | MMRM | Change from baseline to week 8 on MADRS | MMRM | Mahableshwarkar 2015(b) [25] |
|  | 11492A | Change from baseline to week 6 on MADRS | LOCF | Change from baseline to week 6 on MADRS | LOCF | Alvarez 2012 [26] |
|  | 11984A | Change from baseline to week 8 on MADRS | LOCF | Change from baseline to week 8 on MADRS | LOCF | Baldwin 2012 [27] |
|  | 13267A | Change from baseline to week 8 on MADRS | MMRM | Change from baseline to week 8 on MADRS | MMRM | Boulenger 2014 [28] |

### **Meta-analytic results**

#### **Primary findings (broad dose inclusion criteria)**

##### **FDA-based meta-analysis**

The forest plot based on data from FDA reviews using broad dose inclusion criteria are shown in S3 Figure. The numerical results generated by Stata are shown below in Table E in S1 Text.

###### **Table E in S1 Text. Numerical results (FDA-based MA; broad dose inclusion criteria)**

. metan g_corrected se_g, label(namevar=studynumber) by(drug) random

Study | ES [95% Conf. Interval] % Weight

---------------------+---------------------------------------------------

desvenlafaxine

223 | 0.098 -0.181 0.377 2.64

306 | 0.340 0.130 0.550 3.49

308 | 0.368 0.150 0.586 3.38

309 | 0.114 -0.141 0.369 2.91

320 | 0.230 -0.026 0.487 2.89

332 | 0.233 0.036 0.430 3.68

333 | 0.357 0.167 0.548 3.78

304_DVX | 0.142 -0.115 0.399 2.89

317_DVX | 0.091 -0.166 0.347 2.89

Sub-total |

D+L pooled ES | 0.242 0.166 0.319 28.53

---------------------+---------------------------------------------------

levomilnacipran

F02695 LP 2 02 | 0.450 0.282 0.619 4.11

LVM-MD-01 | 0.312 0.140 0.484 4.06

LVM-MD-02 | 0.122 -0.086 0.331 3.52

LVM-MD-03 | 0.271 0.081 0.461 3.78

LVM-MD-10 | 0.305 0.128 0.483 3.98

Sub-total |

D+L pooled ES | 0.302 0.203 0.401 19.45

---------------------+---------------------------------------------------

vilazodone

245 | -0.010 -0.251 0.230 3.08

246 | 0.104 -0.143 0.352 3.00

247 | 0.148 -0.117 0.413 2.79

248 | 0.031 -0.212 0.274 3.05

CLDA-07-DP-02 | 0.243 0.061 0.426 3.89

GNSC-04-DP-02 | 0.332 0.134 0.530 3.66

Sub-total |

D+L pooled ES | 0.158 0.050 0.266 19.48

---------------------+---------------------------------------------------

vortioxetine

303 | 0.069 -0.094 0.232 4.20

305 | 0.513 0.307 0.720 3.54

315 | 0.202 0.006 0.398 3.69

316 | 0.286 0.091 0.480 3.72

11492A | 0.550 0.312 0.789 3.11

11984A | 0.164 -0.034 0.362 3.67

13267A | 0.656 0.459 0.854 3.67

304_VOR | 0.064 -0.162 0.290 3.28

317_VOR | 0.050 -0.148 0.248 3.66

Sub-total |

D+L pooled ES | 0.281 0.130 0.431 32.54

---------------------+---------------------------------------------------

Overall |

D+L pooled ES | 0.243 0.183 0.304 100.00

---------------------+---------------------------------------------------

Test(s) of heterogeneity:

Heterogeneity degrees of

statistic freedom P I-squared** Tau-squared

desvenlafaxine 7.47 8 0.487 0.0% 0.0000

levomilnacipran 5.93 4 0.204 32.6% 0.0042

vilazodone 6.90 5 0.228 27.5% 0.0050

vortioxetine 40.70 8 0.000 80.3% 0.0424

Overall 66.42 28 0.000 57.8% 0.0155

** I-squared: the variation in ES attributable to heterogeneity)

Note: between group heterogeneity not calculated;

only valid with inverse variance method

Significance test(s) of ES=0

desvenlafaxine z= 6.20 p = 0.000

levomilnacipran z= 5.97 p = 0.000

vilazodone z= 2.87 p = 0.004

vortioxetine z= 3.66 p = 0.000

Overall z= 7.93 p = 0.000

-------------------------------------------------------------------------

##### **Journal-based meta-analysis**

The forest plot based on data from journal articles using broad dose inclusion criteria are shown in S4 Figure. The numerical results generated by Stata are shown below in Table F in S1 Text.

###### **Table F in S1 Text. Numerical results (journal-based MA; broad dose inclusion criteria)**

. metan g_corr se_corr, label(namevar=study) by(drug) random

Study | ES [95% Conf. Interval] % Weight

---------------------+---------------------------------------------------

desvenlafaxine

304_DVX | 0.142 -0.115 0.399 3.44

306 | 0.339 0.129 0.549 4.21

308 | 0.368 0.151 0.586 4.07

309&317 | 0.353 0.171 0.535 4.75

320 | 0.230 -0.026 0.487 3.44

332 | 0.244 0.047 0.441 4.46

333 | 0.372 0.181 0.563 4.58

Sub-total |

D+L pooled ES | 0.306 0.227 0.386 28.95

---------------------+---------------------------------------------------

levomilnacipran

F02695 LP 202 | 0.452 0.283 0.621 5.03

LVM-MD-01 | 0.269 0.097 0.440 4.97

LVM-MD-02 | 0.122 -0.086 0.331 4.24

LVM-MD-03 | 0.271 0.081 0.461 4.59

LVM-MD-10 | 0.305 0.128 0.482 4.85

Sub-total |

D+L pooled ES | 0.293 0.192 0.394 23.68

---------------------+---------------------------------------------------

vilazodone

CLDA-07-DP-02 | 0.243 0.061 0.426 4.74

GNSC-04-DP-02 | 0.332 0.134 0.530 4.44

Sub-total |

D+L pooled ES | 0.284 0.150 0.419 9.17

---------------------+---------------------------------------------------

vortioxetine

303 | 0.069 -0.094 0.232 5.15

304_VOR | 0.064 -0.162 0.290 3.94

305 | 0.542 0.325 0.758 4.10

315 | 0.225 0.008 0.442 4.08

316 | 0.310 0.100 0.519 4.23

317 | 0.055 -0.162 0.271 4.10

11492A | 0.571 0.332 0.810 3.71

11984A | 0.164 -0.034 0.362 4.44

13267A | 0.650 0.453 0.848 4.45

Sub-total |

D+L pooled ES | 0.292 0.137 0.447 38.20

---------------------+---------------------------------------------------

Overall |

D+L pooled ES | 0.292 0.228 0.356 100.00

---------------------+---------------------------------------------------

Test(s) of heterogeneity:

Heterogeneity degrees of

statistic freedom P I-squared** Tau-squared

desvenlafaxine 3.41 6 0.756 0.0% 0.0000

levomilnacipran 6.12 4 0.190 34.7% 0.0046

vilazodone 0.42 1 0.519 0.0% 0.0000

vortioxetine 40.62 8 0.000 80.3% 0.0452

Overall 50.86 22 0.000 56.7% 0.0136

** I-squared: the variation in ES attributable to heterogeneity)

Note: between group heterogeneity not calculated;

only valid with inverse variance method

Significance test(s) of ES=0

desvenlafaxine z= 7.54 p = 0.000

levomilnacipran z= 5.69 p = 0.000

vilazodone z= 4.15 p = 0.000

vortioxetine z= 3.68 p = 0.000

Overall z= 8.97 p = 0.000

#### **Sensitivity analysis (narrow dose inclusion criteria)**

##### **FDA-based meta-analysis**

The forest plot based on data from FDA reviews using narrow dose inclusion criteria are shown in S5 Figure. The numerical results generated by Stata are shown below in Table G in S1 Text.

###### **Table G in S1 Text. Numerical results (FDA-based MA; narrow dose inclusion criteria)**

. metan g_corrected se_g, label(namevar=study_number) by(drug) random

Study | ES [95% Conf. Interval] % Weight

---------------------+---------------------------------------------------

desvenlafaxine

332 | 0.269 0.042 0.497 7.20

333 | 0.321 0.102 0.540 7.54

Sub-total |

D+L pooled ES | 0.296 0.138 0.454 14.74

---------------------+---------------------------------------------------

levomilnacipran

F02695 LP 2 02 | 0.450 0.282 0.619 9.96

LVM-MD-01 | 0.312 0.140 0.484 9.80

LVM-MD-02 | 0.122 -0.086 0.331 7.99

LVM-MD-03 | 0.271 0.081 0.461 8.84

LVM-MD-10 | 0.305 0.128 0.483 9.50

Sub-total |

D+L pooled ES | 0.302 0.203 0.401 46.11

---------------------+---------------------------------------------------

vilazodone

CLDA-07-DP-02 | 0.243 0.061 0.426 9.21

GNSC-04-DP-02 | 0.332 0.134 0.530 8.46

Sub-total |

D+L pooled ES | 0.284 0.150 0.419 17.67

---------------------+---------------------------------------------------

vortioxetine

315 | 0.263 0.036 0.491 7.20

316 | 0.357 0.130 0.584 7.21

13267A | 0.739 0.509 0.970 7.07

Sub-total |

D+L pooled ES | 0.453 0.169 0.736 21.48

---------------------+---------------------------------------------------

Overall |

D+L pooled ES | 0.329 0.252 0.407 100.00

---------------------+---------------------------------------------------

Test(s) of heterogeneity:

Heterogeneity degrees of

statistic freedom P I-squared** Tau-squared

desvenlafaxine 0.10 1 0.749 0.0% 0.0000

levomilnacipran 5.93 4 0.204 32.6% 0.0042

vilazodone 0.42 1 0.519 0.0% 0.0000

vortioxetine 9.27 2 0.010 78.4% 0.0493

Overall 19.88 11 0.047 44.7% 0.0083

** I-squared: the variation in ES attributable to heterogeneity)

Note: between group heterogeneity not calculated;

only valid with inverse variance method

Significance test(s) of ES=0

desvenlafaxine z= 3.68 p = 0.000

levomilnacipran z= 5.97 p = 0.000

vilazodone z= 4.15 p = 0.000

vortioxetine z= 3.13 p = 0.002

Overall z= 8.32 p = 0.000

-------------------------------------------------------------------------

##### **Journal-based meta-analysis**

The forest plot based on data from journal articles using narrow dose inclusion criteria are shown in S6 Figure. The numerical results generated by Stata are shown below in Table H in S1 Text.

###### **Table H in S1 Text. Numerical results (Journal-based MA; narrow dose inclusion criteria)**

. metan g_corr se_corr, label(namevar=study) by(drug) random

Study | ES [95% Conf. Interval] % Weight

---------------------+---------------------------------------------------

Desvenlafaxine

332 | 0.274 0.047 0.501 7.33

333 | 0.345 0.126 0.564 7.67

Sub-total |

D+L pooled ES | 0.311 0.153 0.469 15.00

---------------------+---------------------------------------------------

Levomilnacipran

F02695 LP 202 | 0.452 0.283 0.621 10.11

LVM-MD-01 | 0.269 0.097 0.440 9.96

LVM-MD-02 | 0.122 -0.086 0.331 8.13

LVM-MD-03 | 0.271 0.081 0.461 8.99

LVM-MD-10 | 0.305 0.128 0.482 9.65

Sub-total |

D+L pooled ES | 0.293 0.192 0.394 46.84

---------------------+---------------------------------------------------

Vilazodone

CLDA-07-DP-02 | 0.243 0.061 0.426 9.35

GNSC-04-DP-02 | 0.332 0.134 0.530 8.60

Sub-total |

D+L pooled ES | 0.284 0.150 0.419 17.95

---------------------+---------------------------------------------------

Vortioxetine

315 | 0.295 0.040 0.549 6.35

316 | 0.386 0.141 0.632 6.66

13267A | 0.733 0.502 0.963 7.21

Sub-total |

D+L pooled ES | 0.475 0.209 0.742 20.21

---------------------+---------------------------------------------------

Overall |

D+L pooled ES | 0.331 0.253 0.410 100.00

---------------------+---------------------------------------------------

Test(s) of heterogeneity:

Heterogeneity degrees of

statistic freedom P I-squared** Tau-squared

Desvenlafaxine    0.19 1 0.660 0.0% 0.0000

Levomilnacipran  6.12 4 0.190 34.7% 0.0046

Vilazodone  0.42 1 0.519 0.0% 0.0000

Vortioxetine  7.21 2 0.027 72.3% 0.0401

Overall 19.88 11 0.047 44.7% 0.0085

** I-squared: the variation in ES attributable to heterogeneity)

Note: between group heterogeneity not calculated;

only valid with inverse variance method

Significance test(s) of ES=0

Desvenlafaxine    z= 3.86 p = 0.000

Levomilnacipran  z= 5.69 p = 0.000

Vilazodone  z= 4.15 p = 0.000

Vortioxetine  z= 3.49 p = 0.000

Overall z= 8.26 p = 0.000

-------------------------------------------------------------------------

## Multivariate meta-analysis

In our meta-analyses, we were unable to use meta-regression to compare FDA-based and journal-based effect size values, due to non-independence between these two sets of values. As an alternative, exploratory approach, we performed multivariate meta-analysis using the R package *mvmeta*.

The multivariate meta-analysis technique enables one to analyze multiple effect sizes from the same study simultaneously, accounting for the dependence between these effect sizes. In this manuscript, most studies contributed both an FDA-based effect size and a journal-based effect size to the multivariate meta-analysis, while unpublished studies contributed only an FDA-based effect size.

We assumed a cohort-specific within-study correlation of effect sizes, setting it at r=0.671 for the older antidepressant trials and at r=0.944 for the newer antidepressant trials on the basis of the observed correlation between FDA and journal effect sizes in our data. The results of the multivariate meta-analyses are shown in S8 Figure and Table I in S1 Text. These results show that the summary effect from the newer antidepressant trials (black oval) are well-centered on the diagonal, suggesting no publication bias, while the summary effect from the older antidepressant trials (blue oval) is not centered on the diagonal, suggesting the presence of publication bias. Sensitivity analyses using a range of correlation coefficients (r=0.5-0.9) yielded similar results.

Table I in S1 Text. Results of the multivariate meta-analyses

|  | FDA | | Journal | |
| --- | --- | --- | --- | --- |
|  | Hedges’ g (95% CI) | p-value | Hedges’ g (95% CI) | p-value |
| Older antidepressants | 0.30 (0.27-0.34) | <0.001 | 0.36 (0.32-0.41) | <0.001 |
| Newer antidepressants | 0.24 (0.17-0.30) | <0.001 | 0.26 (0.19-0.32) | <0.001 |

*Implications of multivariate meta-analytic results*

Multivariate meta-analysis uses the available effect sizes from a study, and the within-study correlation among effect sizes, to infer what the missing effect sizes “should be”. Thus multivariate meta-analyses can enable researchers to leverage available information (e.g. a specific depression-related outcome in a particular study) to infer something about a missing outcome (e.g. a different depression-related outcome in a particular study). Given a sufficiently high correlation, results for one outcome can provide a good estimate of the results for a missing outcome.

However, in this case, multivariate meta-analysis uses information from the (completely available) FDA-based effect sizes to infer what the journal-based effect size “should be” for unpublished studies. But within the journal dataset, data are not missing at random. Rather, the data from unpublished trials are, with only one exception, statistically nonsignificant. This explains why the journal-based summary effect from the multivariate analysis (S5 Table), compared to the effect from the univariate meta-analysis (main text), comes closer to the FDA-based summary effect. Put another way, the multivariate approach, compared to the univariate approach, diminishes effect size inflation (ESI), the gap between FDA- and journal-based values.

Consequently, the multivariate meta-analysis technique results in an estimate of the journal-based effect size *if there were no systematic study publication bias,* i.e. if non-publication occurred at random. Any gaps between the FDA-based and journal-based summary effect sizes, therefore, are due to the presence of outcome reporting bias. Consequently, the results of these analyses are best interpreted as an indication of the extent of outcome reporting bias specifically, *after accounting for study publication bias,* rather than of reporting bias in general.

Despite these limitations, the results of this approach are consistent with the results presented in the main text, in that the magnitude of reporting bias appears to be less for the newer, as compared to the older, cohort of antidepressant drugs.

## S1 Text References

1. Leitgöb H. The Problem of Modeling Rare Events in ML-based Logistic Regression - Assessing Potential Remedies via MC Simulations. Ljubljana, Slovenia; 2013. Available: https://www.researchgate.net/publication/269708531_The_Problem_of_Modeling_Rare_Events_in_ML-based_Logistic_Regression_-_Assessing_Potential_Remedies_via_MC_Simulations

2. Heinze G, Schemper M. A solution to the problem of separation in logistic regression. Stat Med. 2002;21: 2409–2419. doi:10.1002/sim.1047

3. Allison P. Logistic Regression for Rare Events. 13 Feb 2012. Available: https://statisticalhorizons.com/logistic-regression-for-rare-events

4. Mehta CR, Patel NR. Exact logistic regression: theory and examples. Stat Med. 1995;14: 2143–2160.

5. Firth D. Bias reduction of maximum likelihood estimates. Biometrika. 1993;80: 27–38. doi:10.1093/biomet/80.1.27

6. Liebowitz MR, Yeung PP, Entsuah R. A randomized, double-blind, placebo-controlled trial of desvenlafaxine succinate in adult outpatients with major depressive disorder. J Clin Psychiatry. 2007;68: 1663–1672.

7. DeMartinis NA, Yeung PP, Entsuah R, Manley AL. A double-blind, placebo-controlled study of the efficacy and safety of desvenlafaxine succinate in the treatment of major depressive disorder. J Clin Psychiatry. 2007;68: 677–688.

8. Septien-Velez L, Pitrosky B, Padmanabhan SK, Germain J-M, Tourian KA. A randomized, double-blind, placebo-controlled trial of desvenlafaxine succinate in the treatment of major depressive disorder. Int Clin Psychopharmacol. 2007;22: 338–347. doi:10.1097/yic.0b013e3281e2c84b

9. Lieberman DZ, Montgomery SA, Tourian KA, Brisard C, Rosas G, Padmanabhan K, et al. A pooled analysis of two placebo-controlled trials of desvenlafaxine in major depressive disorder. Int Clin Psychopharmacol. 2008;23: 188–197. doi:10.1097/yic.0b013e32830263de

10. Feiger AD, Tourian KA, Rosas GR, Padmanabhan SK. A placebo-controlled study evaluating the efficacy and safety of flexible-dose desvenlafaxine treatment in outpatients with major depressive disorder. CNS Spectr. 2009;14: 41–50.

11. Liebowitz MR, Manley AL, Padmanabhan SK, Ganguly R, Tummala R, Tourian KA. Efficacy, safety, and tolerability of desvenlafaxine 50 mg/day and 100 mg/day in outpatients with major depressive disorder. Curr Med Res Opin. 2008;24: 1877–1890. doi:10.1185/03007990802161923

12. Boyer P, Montgomery S, Lepola U, Germain J-M, Brisard C, Ganguly R, et al. Efficacy, safety, and tolerability of fixed-dose desvenlafaxine 50 and 100 mg/day for major depressive disorder in a placebo-controlled trial. Int Clin Psychopharmacol. 2008;23: 243–253. doi:10.1097/yic.0b013e32830cebed

13. Montgomery SA, Mansuy L, Ruth A, Bose A, Li H, Li D. Efficacy and safety of levomilnacipran sustained release in moderate to severe major depressive disorder: a randomized, double-blind, placebo-controlled, proof-of-concept study. J Clin Psychiatry. 2013;74: 363–369. doi:10.4088/jcp.12m08141

14. Asnis GM, Bose A, Gommoll CP, Chen C, Greenberg WM. Efficacy and safety of levomilnacipran sustained release 40 mg, 80 mg, or 120 mg in major depressive disorder: a phase 3, randomized, double-blind, placebo-controlled study. J Clin Psychiatry. 2013;74: 242–248. doi:10.4088/jcp.12m08197

15. Gommoll CP, Greenberg WM, Chen C. A randomized, double-blind, placebo-controlled study of flexible doses of levomilnacipran ER (40-120 mg/day) in patients with major depressive disorder. J Drug Assess. 2014;3: 10–19. doi:10.3109/21556660.2014.884505

16. Sambunaris A, Bose A, Gommoll CP, Chen C, Greenberg WM, Sheehan DV. A Phase III, Double-Blind, Placebo-Controlled, Flexible-Dose Study of Levomilnacipran Extended-Release in Patients With Major Depressive Disorder. J Clin Psychopharmacol. 2014;34: 47–56. doi:10.1097/jcp.0000000000000060

17. Bakish D, Bose A, Gommoll C, Chen C, Nunez R, Greenberg WM, et al. Levomilnacipran ER 40 mg and 80 mg in patients with major depressive disorder: a phase III, randomized, double-blind, fixed-dose, placebo-controlled study. J Psychiatry Neurosci. 2014;39: 40–49. doi:10.1503/jpn.130040

18. Khan A, Cutler AJ, Kajdasz DK, Gallipoli S, Athanasiou M, Robinson DS, et al. A randomized, double-blind, placebo-controlled, 8-week study of vilazodone, a serotonergic agent for the treatment of major depressive disorder. J Clin Psychiatry. 2011;72: 441–447. doi:10.4088/jcp.10m06596

19. Rickels K, Athanasiou M, Robinson DS, Gibertini M, Whalen H, Reed CR. Evidence for efficacy and tolerability of vilazodone in the treatment of major depressive disorder: a randomized, double-blind, placebo-controlled trial. J Clin Psychiatry. 2009;70: 326–333.

20. Jain R, Mahableshwarkar AR, Jacobsen PL, Chen Y, Thase ME. A randomized, double-blind, placebo-controlled 6-wk trial of the efficacy and tolerability of 5 mg vortioxetine in adults with major depressive disorder. Int J Neuropsychopharmacol. 2013;16: 313–321. doi:10.1017/s1461145712000727

21. Mahableshwarkar AR, Jacobsen PL, Chen Y. A randomized, double-blind trial of 2.5 mg and 5 mg vortioxetine (Lu AA21004) versus placebo for 8 weeks in adults with major depressive disorder. Curr Med Res Opin. 2013;29: 217–226. doi:10.1185/03007995.2012.761600

22. Henigsberg N, Mahableshwarkar AR, Jacobsen P, Chen Y, Thase ME. A randomized, double-blind, placebo-controlled 8-week trial of the efficacy and tolerability of multiple doses of Lu AA21004 in adults with major depressive disorder. J Clin Psychiatry. 2012;73: 953–959. doi:10.4088/jcp.11m07470

23. Mahableshwarkar AR, Jacobsen PL, Chen Y, Serenko M, Trivedi MH. A randomized, double-blind, duloxetine-referenced study comparing efficacy and tolerability of 2 fixed doses of vortioxetine in the acute treatment of adults with MDD. Psychopharmacology (Berl). 2015;232: 2061–2070. doi:10.1007/s00213-014-3839-0

24. Jacobsen PL, Mahableshwarkar AR, Serenko M, Chan S, Trivedi MH. A randomized, double-blind, placebo-controlled study of the efficacy and safety of vortioxetine 10 mg and 20 mg in adults with major depressive disorder. J Clin Psychiatry. 2015;76: 575–582. doi:10.4088/jcp.14m09335

25. Mahableshwarkar AR, Jacobsen PL, Serenko M, Chen Y, Trivedi MH. A randomized, double-blind, placebo-controlled study of the efficacy and safety of 2 doses of vortioxetine in adults with major depressive disorder. J Clin Psychiatry. 2015;76: 583–591. doi:10.4088/jcp.14m09337

26. Alvarez E, Perez V, Dragheim M, Loft H, Artigas F. A double-blind, randomized, placebo-controlled, active reference study of Lu AA21004 in patients with major depressive disorder. Int J Neuropsychopharmacol. 2012;15: 589–600. doi:10.1017/s1461145711001027

27. Baldwin DS, Loft H, Dragheim M. A randomised, double-blind, placebo controlled, duloxetine-referenced, fixed-dose study of three dosages of Lu AA21004 in acute treatment of major depressive disorder (MDD). Eur Neuropsychopharmacol. 2012;22: 482–491. doi:10.1016/j.euroneuro.2011.11.008

28. Boulenger J-P, Loft H, Olsen CK. Efficacy and safety of vortioxetine (Lu AA21004), 15 and 20 mg/day: a randomized, double-blind, placebo-controlled, duloxetine-referenced study in the acute treatment of adult patients with major depressive disorder. Int Clin Psychopharmacol. 2014;29: 138–149. doi:10.1097/yic.0000000000000018
